# Cases in Practice

**Published:** 1858-04

**Authors:** E. Read

**Affiliations:** Terre Haute, Ind.


### THE CHICAGO MEDICAL JOURNAL.

VOL. I. APRIL, 1858. No. 4.

### ORIGINAL COMMUNICATIONS.

### ARTICLE I.

### TWO CASES OF ABSCESS OF THE LUNGS; CERVICAL SUPPURATION WITH HÆMORRHAGE, A DANGEROUS COMPLICATION OF *SCARLATINA; PLACENTA PRÆVIA*, A CASE OF TURNING AND DELIVERY, WITH SAFETY TO MOTHER AND CHILD.

BY E. READ, M.D., TERRE HAUTE, IND.

In the summer of 1844, I visited a patient, eighteen miles distant, who was represented to have a very bad cough and to be expectorating large quantities of pus. On my arrival, I found a man, aged twenty-seven years, who had always enjoyed good health up to within two months of that time, and whose occupation was that of a farmer. I found the left side protuberant and swollen, and puffed out, from the internal pressure of matter.

He was expectorating pus very freely, and had exacerbations of fever attended with night sweats, and had every appearance of one rapidly declining with consumption. His own history, for he had not had any medical attention, living as he did remote from towns, was that, two months previously, he had taken a cold which was accompanied with a severe pain in the left side, which had continued up to within a few days of the time that I saw him. He could not lie upon his back or right side. It seemed as though the entire left lung was converted into pus, and I recommended, for I could see nothing else to do, the operation for empyema. He had a most terrible apprehension of the puncture, and would not consent to the operation under any circumstances whatever, declaring he would rather die without it, than to live with it.

Having prescribed expectorants and anodynes, I left him with a full conviction in my own mind that he would certainly die within a very short time.

A month afterwards, I saw one of his neighbors, who informed me that the tumor had burst externally just below the left nipple, and had discharged a great quantity of matter and was still discharging, and that the man was better. The next winter I understood that he was making rails and chopping wood, and had actually engaged to clear off a heavily-timbered piece of land, although his side was still discharging matter. In the summer of 1855, eleven years from the time I first saw him, he came to see me. Upon examination, I found a thin discharge was still kept up, and that all of the ribs of the left side were externally concave, instead of convex. The left lung was entirely gone, and that whole side had the appearance of being pushed in. He informed me that he had enjoyed good health, and worked daily at the hardest and most exposed labor of the farm. In every respect he seemed robust and sufficiently healthy, and thought that he had his usual strength and weight. He was still living a year ago.

*Abscess of the Lungs, opening into the Bronchial Tubes.*—In the month of March, 1856, I was summoned in great haste to see J. F. K., who was represented to be suffocating from the discharge of pus from his lungs. In twenty minutes' time I was with him, and never have I seen so large a quantity of pus thrown out in so short a time. He was sitting strangling, coughing and almost suffocating, and had already filled a tin basin holding three pints, and was now filling a second, and continued until it was quite full. The stream was continuous, and nearly an inch in diameter. The first basin of matter was pure yellow pus, the second, after being half filled with the pure pus, became streaked with blood. Here, then, in a little over half an hour, the very large quantity of pus above mentioned was discharged *per orem*.

The subject of this abscess was a large fleshy man, aged sixty-five years, who had always enjoyed most excellent health, although his respiration was a little hurried, and he generally seemed short of breath. For a few weeks previous to this, he had had a fixed pain in his left side, but not severe enough to call for medical aid, and but a few minutes before the present attack, he had been working in the yard among his fruit trees. Immediately after he went into the house, he commenced coughing, and the discharge of pus followed. He remained in-doors but two or three days, and then went out as usual. During the summer he enjoyed his usual health, but in the next October he began to cough, which was attended with a suffocating sensation, but without pain, and gradually he lost flesh, and was unable to rest in any other than the sitting posture. Anasarca supervened, and he died in January. The abscess seemed to have involved almost the entire left lung. I could not obtain the consent of the family to make a post-mortem examination.

*Scarlatina, and one of its Dangerous Complications.*—A few years ago, in consultation, I saw a little girl, aged eight years, who, as a sequent or rather as a concomitant of scarlatina, had swelled submaxillary glands, which terminated in suppuration, and involved the entire cellular tissue of one side and front of the neck. The attending physician lanced the abscess, and after a large quantity of matter was discharged, bleeding ensued, which continued for three days, when she died of exhaustion from the loss of blood.

In every other respect she appeared to be doing well, and would unquestionably have recovered had it not been for the submaxillary and submental suppuration, which destroyed so many of the bloodvessels, that a fatal hemorrhage ensued.

Every possible means was resorted to, to arrest the bleeding. The blood was thin and pale, and seemed destitute of fibrin and coloring matter and albumen. The family never forgave the physician, for they believe that from anatomical ignorance, he had cut a bloodvessel, which he was unable to secure. Since then, I have seen two others bleed to death from the same cause. They were both my own patients, and as I had received a very salutary lesson from the first one I saw, I carefully abstained from the use of the lancet. I left to Nature both to open the abscess and receive the censure.

I am persuaded that most will die from hemorrhage, where the suppuration is extensive in this region; hence, the safest plan for the physician is to avoid all risks of censure, especially as the delay to open the abscess will in no way add to the danger of the patient. The last case I saw was but a few days ago, and was that of a boy six years of age, who died on the fourteenth day of his sickness, and on the fourth of the hemorrhage.

The cervical suppuration may be regarded as a dangerous complication of scarlatina, because it gives rise to a hemorrhage which is exceedingly unmanageable. Just now, *scarlatina anginosa* is prevailing here, with an unusual disposition to cervical swellings, although I have seen but one which terminated in suppuration. Nitrate of silver, xx grs. to the oz. of water, seems almost a specific as a local application to the throat internally. It should be thoroughly applied two or three times a day.

In the month of December, there were but three interments in the city of Terre Haute, which shows one of two things—either that we have an unusually healthy locality, or that our physicians are well skilled in the healing art. If the scarlet fever, however, continues to prevail, the next monthly report of interments will probably be largely increased.

*Placenta Prœvia.*—I saw an interesting case of placenta prævia yesterday, in consultation with Dr. Clippinger of this city, which, by promptly turning, terminated thus far safely both to mother and child.

It was the fourth child of a healthy mother. Having been in labor twenty-four hours, Dr. C. was called, who found, upon examination, the membranes protruding, with a well-dilated os uteri. Upon the first pain after his arrival they were ruptured, which revealed the placenta filling up the os uteri. As there was already some hemorrhage, I was called in consultation, and upon examination, found as Dr. C. described, a presenting placenta. Above and just under the pelvic arch, the head was presenting. The placenta was partially adherent to the posterior part of the neck of the uterus. We decided an immediate turning and delivery, and the patient being placed in proper position, Dr. C. introduced his hand, and in a very skillful manner grasped the feet and delivered the child in five minutes' time. The child was resuscitated with a good deal of difficulty, but to-day seems quite well and healthy. The woman lost but little blood, and has every prospect of a speedy recovery. Would not a less prompt practice have endangered the life of both mother and child?

From all that I have read, and all that I have seen, I am persuaded that no physician should hesitate a moment, in all cases of placenta prævia, to turn and deliver in the most prompt manner. In no other, are delays so dangerous. Death may ensue from a single gush of blood in these. It should then, I think, be laid down as an axiom in obstetrical practice, to deliver without delay in all cases of *placenta prævia.*

